# The geographical distribution of Burkitt's tumour compared with the geographical distribution of other types of malignant lymphoma in Uganda.

**DOI:** 10.1038/bjc.1966.57

**Published:** 1966-09

**Authors:** D. H. Wright, M. Roberts

## Abstract

**Images:**


### THE GEOGRAPHICAL DISTRIBUTION OF BURKITT'S TUMOUR COMPARED WITH THE GEOGRAPHICAL DISTRIBUTION OF OTHER TYPES OF MALIGNANT LYMPHOMA IN UGANDA

D. H. WRIGHT AND M. ROBERTS

*From the Department of Pathology, Makerere University College Medical School, Kampala, Uganda*

Received for publication, April 25, 1966

THE distribution of Burkitt's tumour in Africa corresponds closely to those areas that have a mean annual minimum temperature of not less than 60° F. and a rainfall of more than 20 inches a year (Burkitt, 1963 ; Haddow, 1963). Uganda which lies astride the Equator varies in altitude from the highest peak of the Mountains of the Moon at over 16,000 feet to barely 2,000 feet where the Albert Nile crosses into the Sudan. The mountainous plateau that forms Rwanda and Burundi extends northwards into south-western Uganda and most of the inhabited areas of this region lies between 5,000 and 7,000 feet above sea level. Burkitt and Wright (1966) have shown that the incidence of Burkitt's tumour is twenty times greater in the low lying areas north and west of the Nile than it is in this densely populated highland area of south-western Uganda. They concluded that this wide variation in incidence is related to differences in altitude and hence to temperature, and that the apparent temperature dependence of Burkitt's tumour within Uganda supports the hypothesis that this tumour may be induced by an insect vectored virus.

This study was undertaken to see whether malignant lymphomas other than Burkitt's tumour show any variation in their geographical distribution within Uganda.

### MATERIALS AND METHODS

The Department of Pathology of Makerere University College Medical School has provided the histopathology services for the whole of Uganda since 1961. The Uganda Government Medical Service was responsible for the histopathology of all hospitals other than Mulago Hospital, Kampala, before 1961. All histologically proven cases of malignant disease in Uganda are recorded in the Kampala Cancer Registry and the sections and paraffin blocks of these cases are filed in the Department of Pathology. In this study the histological sections of all cases of Burkitt's tumour and all other types of malignant lymphoma biopsied in Uganda during the period 1959 to 1964 inclusive were reviewed and classified.

The classification used was based on that proposed by Gall and Rappaport (1958) and Rappaport (1963). The criteria used in this department for the identification of Burkitt's tumour are briefly as follows :

### *Cytology* (Wright, 1963) (Fig. 1)

Giemsa stained imprint preparations of tumour were made from almost all cases of Burkitt's tumour biopsied at Mulago Hospital since 1961. In these preparations the cells of Burkitt's tumour have a characteristic morphology, different from that of any other malignant lymphoma. They range in size from 20 to 30 $\mu$. Their nuclei are round, oval, deeply cleft or trefoil in shape with a finely stippled nuclear chromatin and two to five inconspicuous nucleoli. The cytoplasm forms a well defined eccentric rim around the nucleus and is intensely basophilic apart from a pale staining area opposite the nuclear indentation. Cytoplasmic vacuoles are a prominent feature of most of these preparations and are invariably present in at least some of the cells. Large clear histiocytes laden with whole cells or pyknotic cell debris are usually found interspersed between the lymphoid cells.

*Histology* (Fig. 2)

Histological sections are subject to a variety of fixation, sectioning and staining artefacts and are less reliable as a means of identifying Burkitt's tumour than cytological preparations. Nevertheless, if the biopsy is well fixed in buffered formalin it is possible to identify Burkitt's tumour in haematoxylin and eosin stained sections and to differentiate it from other types of malignant lymphoma.

The tumour is composed of uniform sheets of primitive lymphoid cells with finely stippled or vesicular nuclear chromatin. The cytoplasm which is rich in ribonucleic acid has an amphophilic staining quality. Careful inspection of this rim of cytoplasm will usually reveal the vacuoles that are such a conspicuous feature of most imprint preparations.

Scattered between the lymphoid cells are large clear or vacuolated non-neoplastic histiocytes. These are usually laden with cells or cell debris and give the so-called " starry sky " pattern to sections of the tumour. This is not a specific feature but is usually much more prominent in Burkitt's tumour than in other types of malignant lymphoma.

*Clinical features*

Although the diagnosis of Burkitt's tumour was based mainly on cytological and histological criteria, clinical features were taken into account in arriving at a final diagnosis in those cases in which cytology was not available.

After classification the cases were plotted on a map of Uganda. Since the exact home address of all cases was not known, they were plotted according to the nearest hospital to their home. This was usually the hospital at which the biopsy was taken although a number of cases biopsied at Mulago Hospital, Kampala, had been referred from " up country " hospitals.

### RESULTS

In 749 cases sufficient information about the patients home address was available and the histological sections were of sufficient quality for acceptance into this study. The histological classification of these cases is shown in Table I. For ease of plotting the cases were condensed into four broad histological groupings.

---

EXPLANATION OF PLATE

FIG. 1.—Imprint of Burkitt's tumour stained with May Grunwald Giemsa. ×2300.
FIG. 2.—Section of Burkitt's tumour stained with haematoxylin and eosin. ×530.

1

2

Wright and Roberts.

TABLE I.—*Histological Classification of* 749 *Cases of Malignant Lymphoma Seen in Uganda from* 1959 *to* 1964 *Inclusive*

| | |
|---|---|
| Histiocytic—lymphoma / Stem cell lymphoma } Reticulum cell sarcoma . . . | 191 |
| Hodgkin's disease (paragranuloma, granuloma and sarcoma) . | 106 |
| Lymphocytic lymphoma (lymphosarcoma) . . . . | 128 |
| Burkitt's tumour . . . . . . . . . . | 324 |
| Total | 749 |

The distribution of the 324 cases of Burkitt's tumour is shown in Fig. 3. The distribution follows the main population centres (Fig. 4) in the north and east of

FIG. 3.—Map of Uganda showing the distribution of 324 cases of Burkitt's tumour biopsied between 1959 and 1964.

the country and around Kampala but not in the densely populated south western region served by Fort Portal, Mbarara, Kabale and Kisoro hospitals. Only 16 cases (5 per cent of the total) were seen in this area which contains 20 per cent of the Uganda population. Mbarara has an altitude of 4,832 feet above sea level and serves the lower lying area towards Lake Victoria as well as the escarpment area to the west. One of the two cases seen at Kisoro was a 20-year-old man who had worked in Kampala for several years and had returned to his home when he fell ill. Six of the eleven cases seen at Mbarara were over the age of 16 years. One of these was an 18-year-old refugee from neighbouring mountainous Rwanda who had entered Uganda two years previously and had lived for part of this time in Masaka.

The distribution of the 425 cases of malignant lymphoma other than Burkitt's

FIG. 4.—Map of Uganda showing the population density in persons per square mile.

tumour shown in Fig. 5. These follow closely the distribution that would be expected on the basis of population density (Fig. 4) and medical facilities. Seventy-six cases (18 per cent of the total) occurred in the south-western region served by Fort Portal, Mbarara and Kabale hospitals.

### DISCUSSION

Burkitt and Wright (1966) have recently made a detailed analysis of the distribution of Burkitt's tumour in Uganda. The cases of Burkitt's tumour reported in this study form part of the larger series analysed by them. Using the popula-

FIG. 5.—Map of Uganda showing the distribution of 425 cases of malignant lymphoma, other than Burkitt's tumour, biopsied between 1959 and 1964.

tion figures of the 1959 Uganda census they showed that Burkitt's tumour is twenty times more common in northern Uganda than it is in the south west of the country. This variation in tumour incidence can be correlated closely with altitude which in turn can be correlated with temperature. The tumour is least common in those areas which have an altitude of greater than 5,000 feet above sea level in which the mean annual minimum temperature falls below 60° F. They also noted that the average age of the cases of Burkitt's tumour was greater in those areas where the tumour was least common than it was in those areas where it was most common.

Burkitt and Wright stated that the geographical variation in incidence of Burkitt's tumour in Uganda could not be due to variations in medical facilities and communications which are at least as good in the south west as in the north of Uganda. The observations reported here on the distribution of malignant lymphomas other than Burkitt's tumour in Uganda support this statement. These tumours show the distribution that would be expected on the basis of population density and medical facilities. They do not show the preponderance of cases in northern Uganda nor the paucity of cases in the south-west that is shown by Burkitt's tumour.

The apparent dependence of the distribution of Burkitt's tumour in Uganda on temperature supports the hypothesis that this tumour may be induced by an arthropod borne virus.

### SUMMARY

The geographical distribution of 324 histologically proven cases of Burkitt's tumour seen in Uganda between 1959 and 1964 inclusive is compared with the geographical distribution of 425 histologically proven malignant lymphomas other than Burkitt's tumour seen in the same period. Whereas Burkitt's tumour shows a marked preponderance of cases north and west of the River Nile and a paucity of cases in south western Uganda, the distribution of all other lymphomas follows the pattern that would be expected on the basis of population density and medical facilities.

We are indebted to Mr. W. Serumaga of the Department of Medical Illustration of Makerere University College for Fig. 3, 4 and 5. This work was made possible by a grant from the British Empire Cancer Campaign for Research, which also supports the Kampala Cancer Registry.

### REFERENCES

Burkitt, D.—(1963) 'Symposium on lymphoreticular Tumours in Africa'. Paris, 1963, p. 119. Edited by F. Roulet. Basel/New York (S. Karger), 1964.

Burkitt, D. and Wright, D. H.—(1966) *Br. med. J.*, i, 569.

Gall, E. A. and Rappaport, H.—(1958) 'Proceedings of seminar on diseases of lymph nodes and spleen'. Chicago (American Society of Clinical Pathologists), 1958.

Haddow, A. J.—(1963) *E. Afr. med. J.*, **40**, 429.

Rappaport, H.—(1963) 'Symposium on lymphoreticular Tumours in Africa'. Paris, 1963. p. 174. Edited by F. Roulet. Basel/New York (S. Karger), 1964.

Wright, D. H.—(1963) *Br. J. Cancer*, **17**, 50.


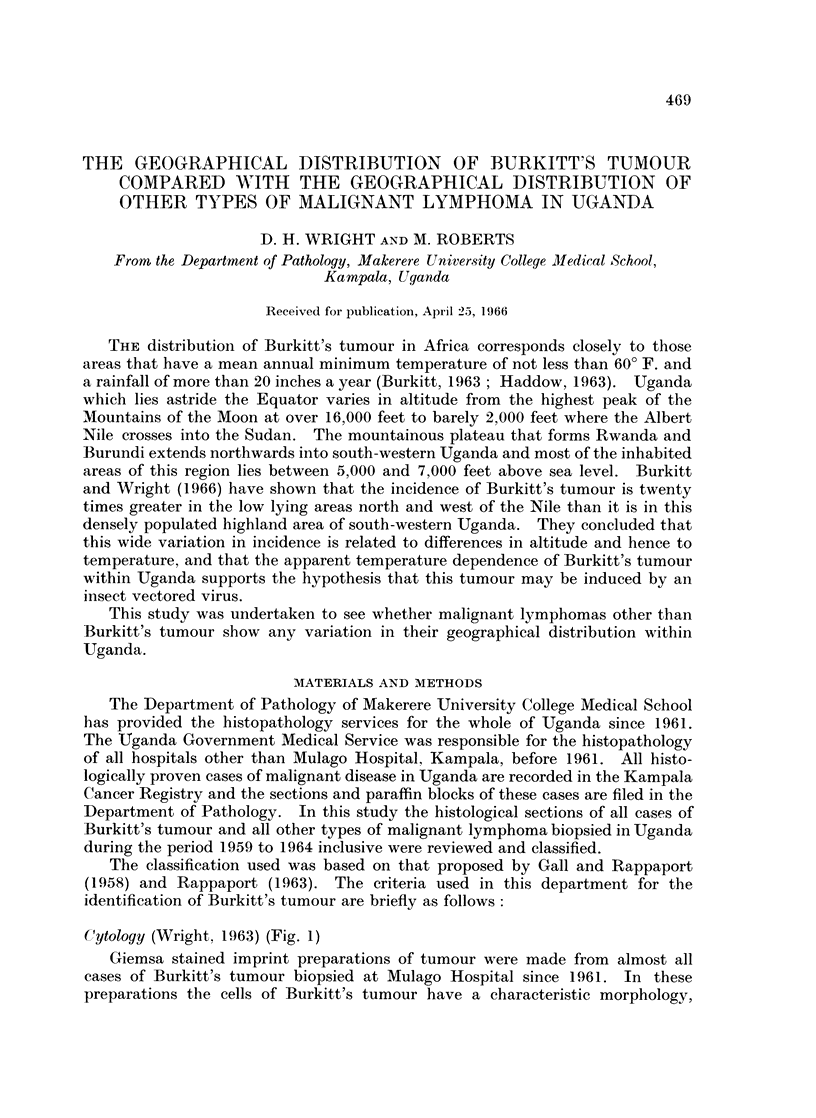

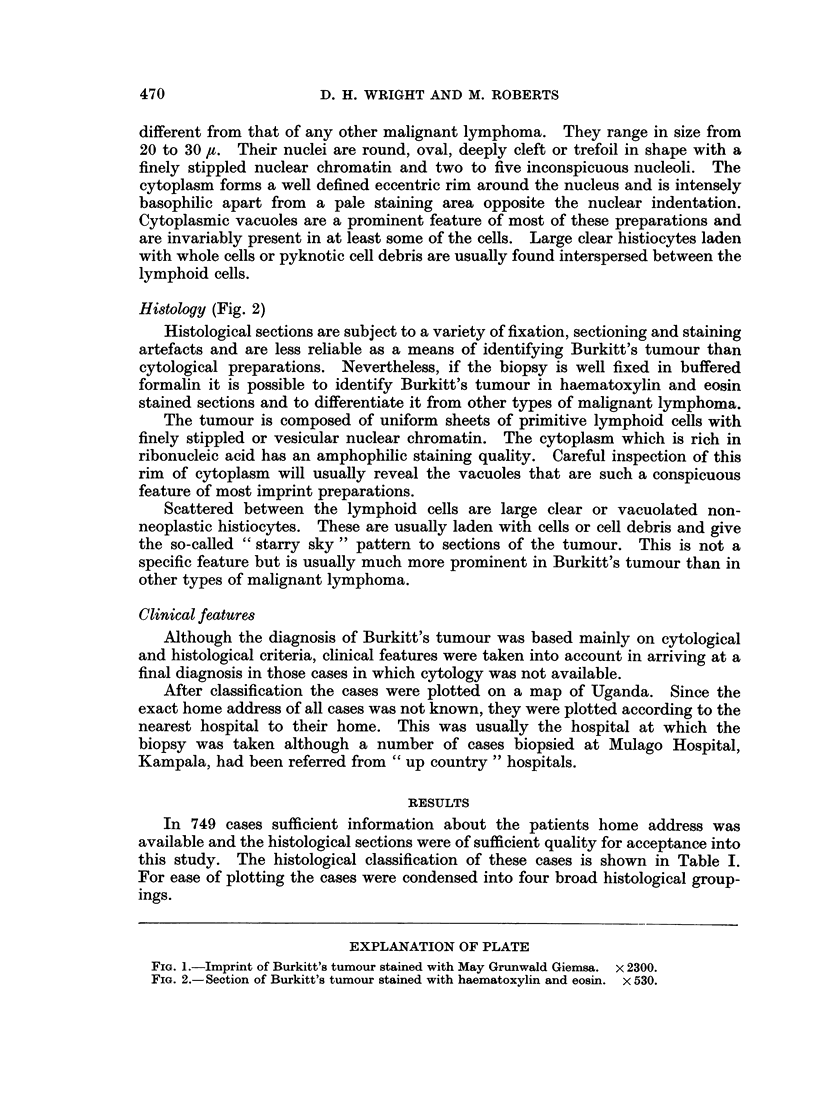

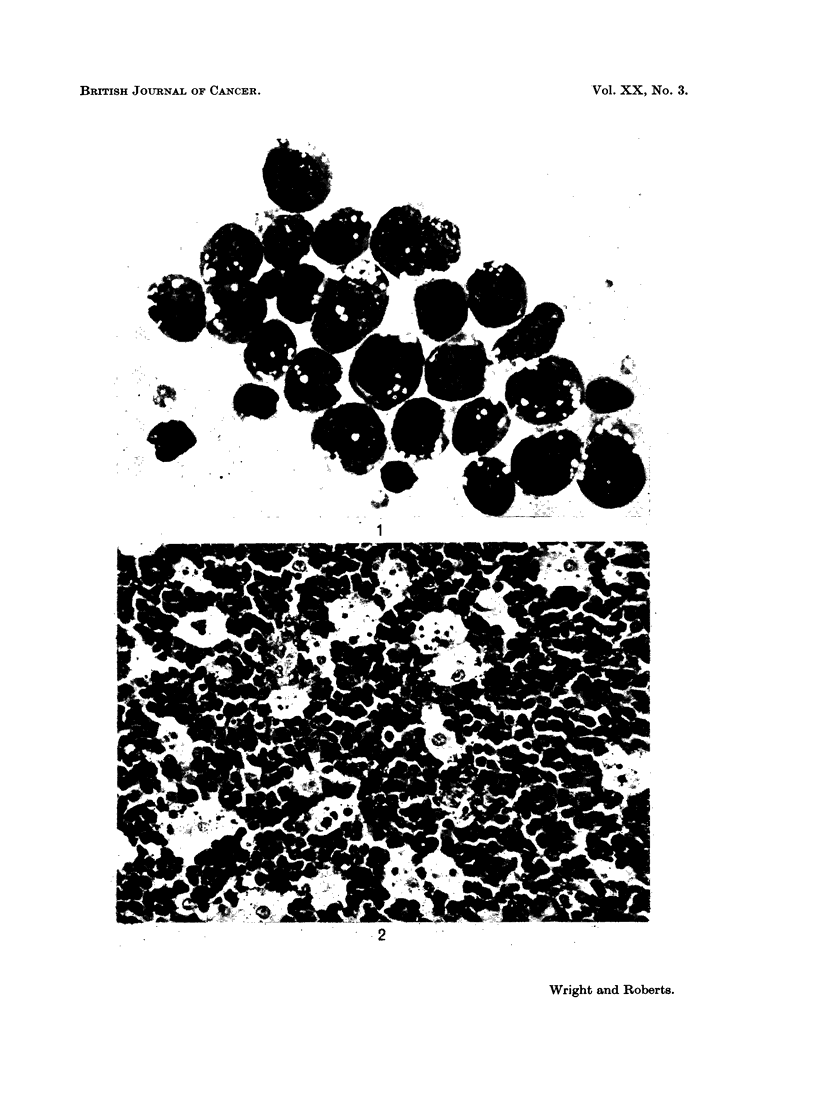

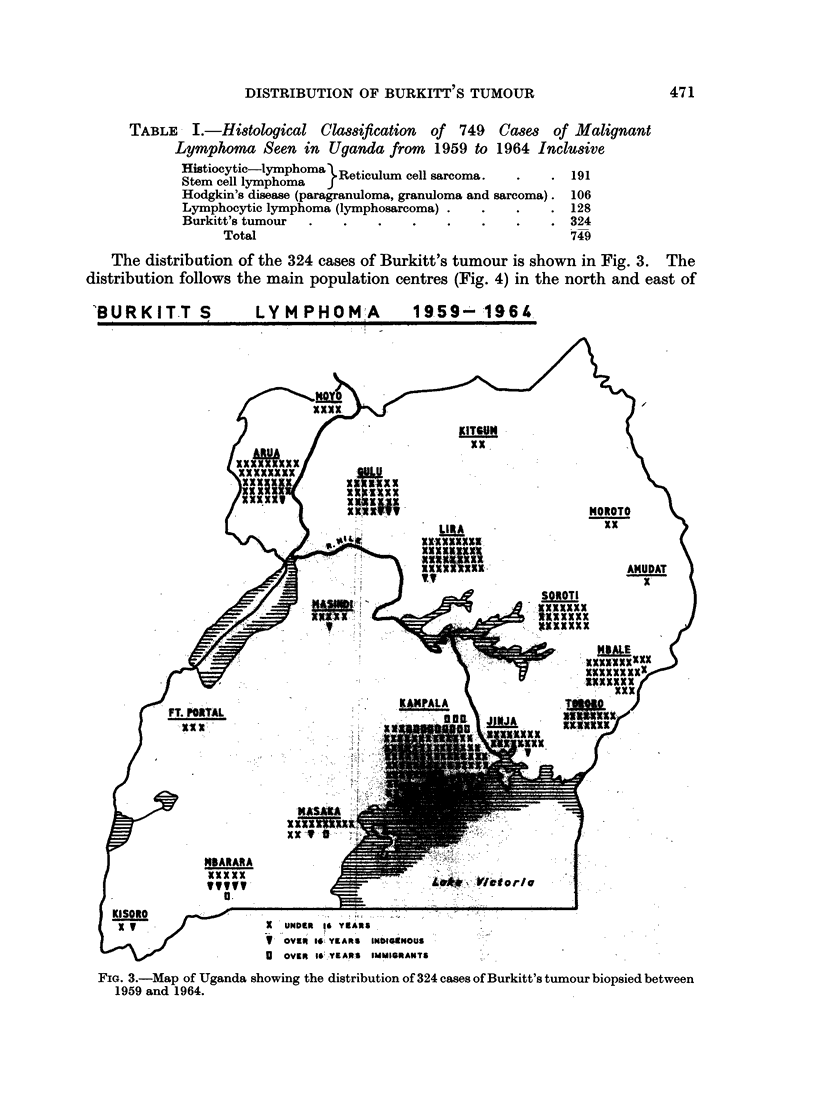

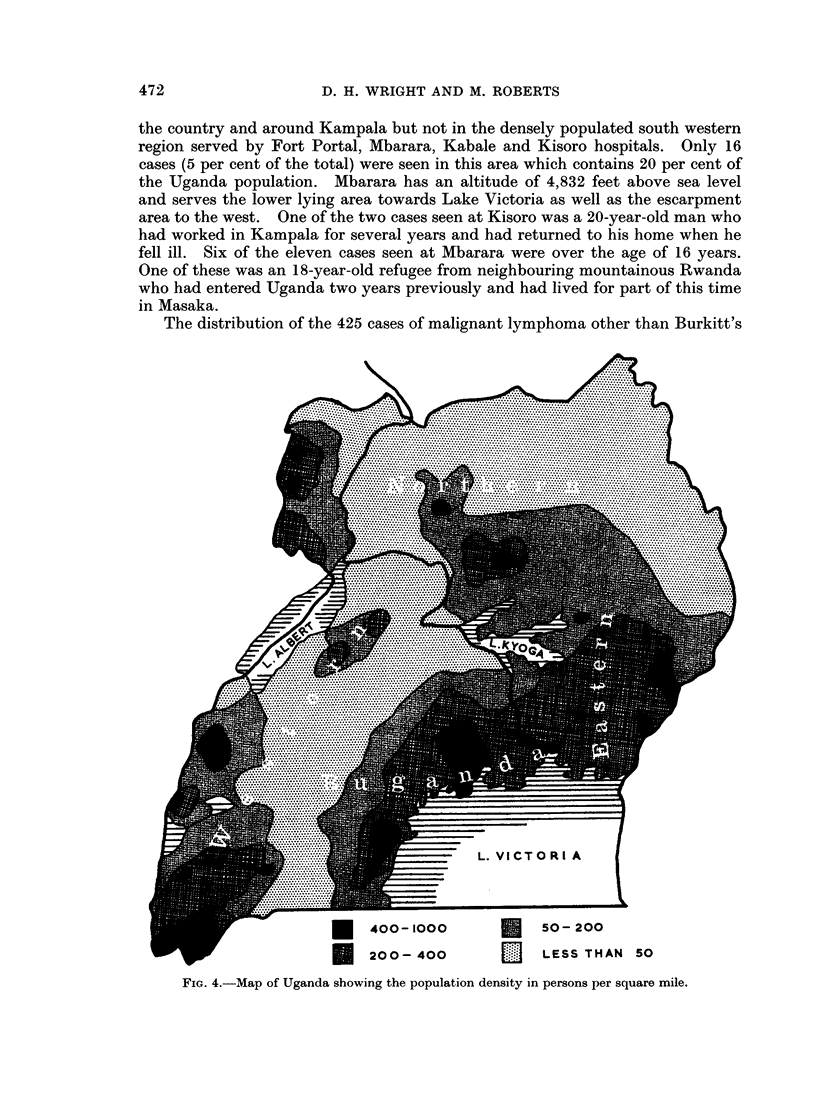

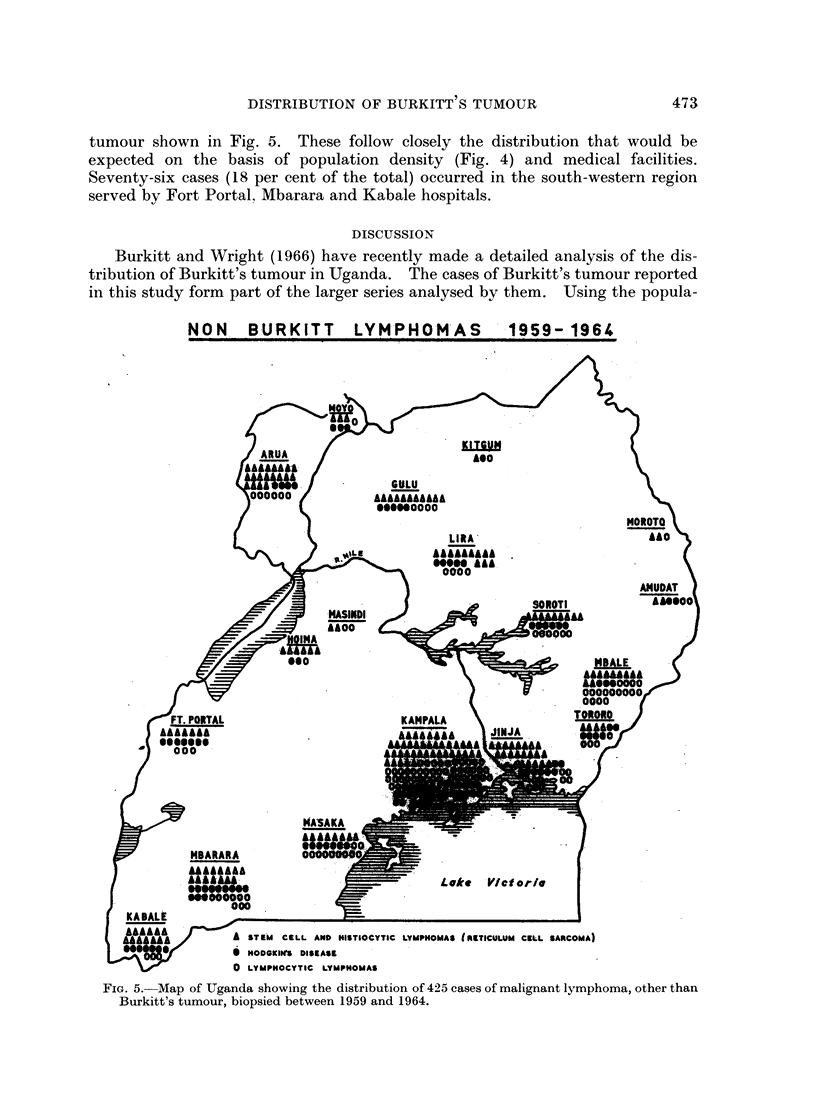

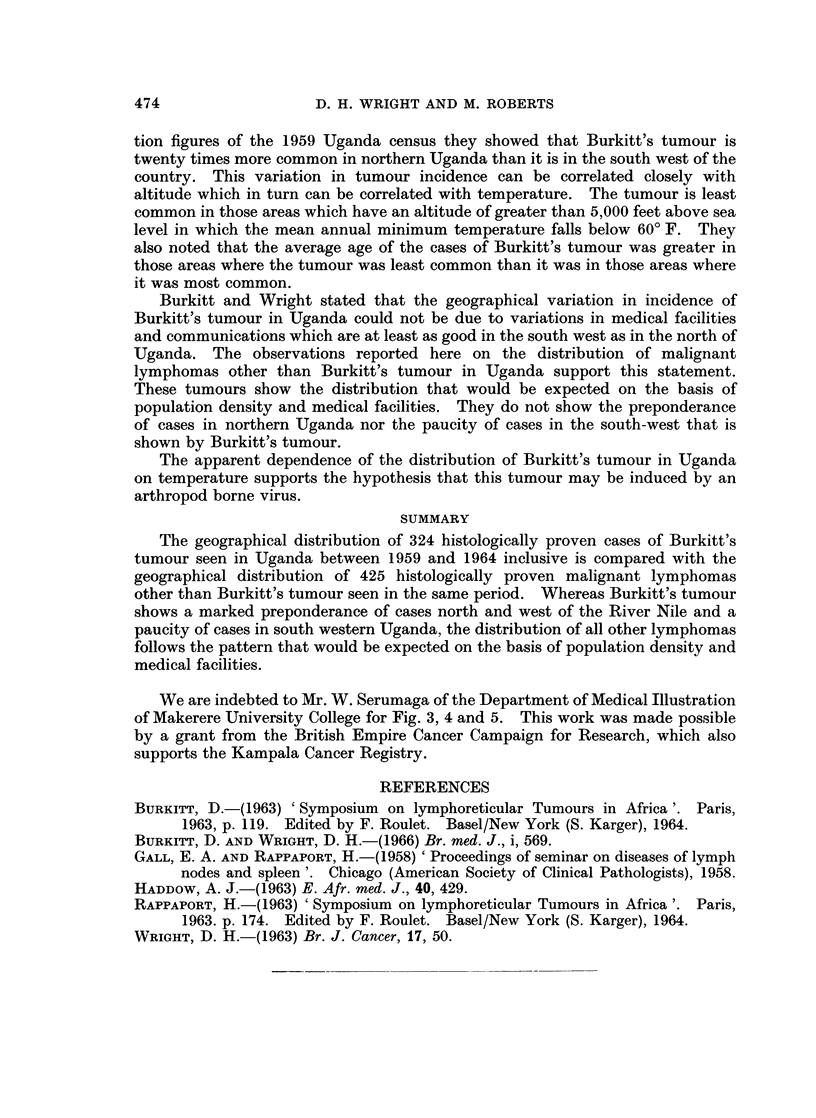


## References

[OCR_00255] Burkitt D., Wright D. (1966). Geographical and tribal distribution of the African lymphoma in Uganda.. Br Med J.

[OCR_00260] HADDOW A. J. (1963). AN IMPROVED MAP FOR THE STUDY OF BURKITT'S LYMPHOMA SYNDROME IN AFRICA.. East Afr Med J.

